# Survivorship Considerations and Management in the Adolescent and Young Adult Sarcoma Population: A Review

**DOI:** 10.3390/curroncol32040214

**Published:** 2025-04-03

**Authors:** Allison Gunderson, Miriam Yun, Babe Westlake, Madeline Hardacre, Nicholas Manguso, Alicia A. Gingrich

**Affiliations:** 1 Reno School of Medicine, University of Nevada, Reno, NV 89557, USA; 2 University of Nevada, Reno, NV 89557, USA; 3 Division of Surgical Oncology, Department of Surgery, University of Nevada, Reno/Renown Integrated Health System, Reno, NV 89502, USA; 4 Division of Breast Surgical Oncology, Department of Surgery, University of Nevada, Reno/Renown Integrated Health System, Reno, NV 89502, USA; madeline.hardacre@renown.org

**Keywords:** soft tissue sarcoma, adolescent young adult, survivorship, early and late secondary effects

## Abstract

Soft tissue sarcoma (STS) has an 2–8% incidence for all malignant tumors in the adolescent and young adult (AYA) population, which are patients from ages 15 to 39. As most STS tumors are aggressive, they require multimodal management with surgery, radiation and chemotherapy. This article discusses the survivorship considerations in this young population of cancer patients who complete therapy. The lasting side effects include surgical and radiation-related morbidity, chemotherapy toxicity, early and late secondary effects on other organ systems, such as cardiac and endocrine dysfunction, and the development of secondary cancers. The long-term psychologic and practical impacts for those who have received a sarcoma diagnosis in the prime of their life include fertility, mental health, relationship, education and career implications. Although there is a paucity of data in some of these areas, we present existing management guidelines as available. This article serves as a comprehensive review of this wide array of treatment effects intended for all providers participating in the care of AYA sarcoma survivors, to include oncologists, primary care providers and therapists.

## 1. Introduction

Soft tissue sarcomas (STSs) are rare mesenchymal neoplasms, which account for approximately 1% of all solid tumors in the general population [1]. However, the incidence reaches 2–8% of all malignant tumors in the adolescent and young adult (AYA) population, defined as patients between the ages of 15 and 39 years [2,3]. STS can occur throughout the body and is comprised of >100 histologic subtypes. The incidence distribution of the histologic subtypes among AYA patients differs from the adult population [3]. The diversity of this family of neoplasms includes many subtypes of high-grade tumors with a propensity for local recurrence and metastasis. Therefore an aggressive, multimodal treatment regimen is often indicated in an effort to improve survival in these young patients [4].

**Figure 1:**
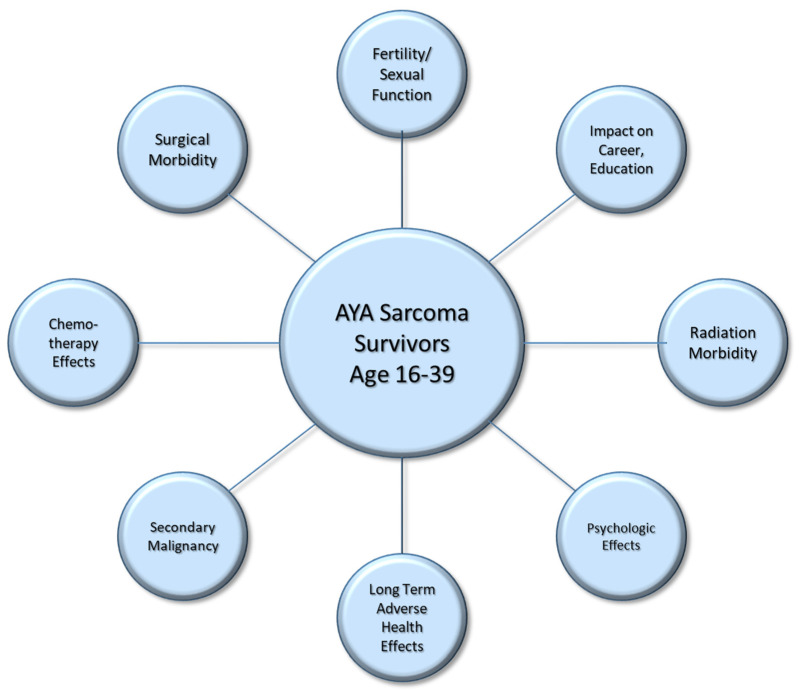
Survivorship considerations for adolescent and young adult patients following a diagnosis of soft tissue sarcoma.

However, receiving such aggressive treatment early in life has ramifications. AYA patients are increasingly being recognized as their own “oncologic age group”, with unique psychosocial characteristics and a greater duration to manage long-term treatment side effects due to the number of life-years remaining [2,5]. Once the primary treatment has concluded, surveillance for recurrent or metastatic disease is obviously paramount. However, the lasting effects of treatment pertain to many other dimensions of their lives as well. In this review, we present survivorship considerations for AYA patients following a diagnosis of soft tissue sarcoma (Figure 1), to include living with surgical morbidity, long-term chemotherapy toxicities and radiation changes. It has also been established that AYA patients are at an increased risk for secondary cancers [6] and adverse health effects on other organ systems, such as cardiac and endocrine dysfunction [7,8,9].

Finally, we discuss the long-term psychologic and social impacts for those who have received a sarcoma diagnosis in the prime of their life. Impacts of the diagnosis extend to their mental health, ability to continue their education and preparation to enter the workforce, advance their career and maintain romantic relationships. The ability to start a family is both physically and emotionally affected by treatment and fertility considerations and must be discussed early [10,11,12]. With a thorough understanding and comprehensive approach, physicians can optimize not only survival outcomes but the quality of their survivorship as well.

## 2. Treatment-Related Late Effects

### 2.1. Long-Term Morbidities from Local Treatment

Surgical interventions are a cornerstone in the treatment of many cancers, particularly in AYA patients, where procedures aim to remove tumors or reduce disease burden. In the case of STS, radiation therapy is often an important adjunct for tumors of the extremity. Given the ability of soft tissue sarcomas to present throughout the body, to include the extremities, abdomen, retroperitoneum and thorax, the long-term morbidity following local treatment varies widely.

Extremity resections include both amputations and limb-sparing surgery (LSS), the latter of which is often paired with radiation (RT). While LSS is preferred in many cases if possible, long-term risks are possible. One of the first studies of patients undergoing LSS with post-operative RT found that 57% of patients had tissue indurations, 20% of patients experienced contractures, 19% had greater than 2+ edema, 7% required narcotics for pain and 6% had fractures. Additionally, 32% had a significant decrease in their range of motion, 20% had a decrease in strength, 16% required an orthotic device or cane and 9% had chronic infections [13]. A subsequent study was conducted more recently and, despite advances in RT techniques, post-operative RT was still associated with late toxicities in 71% of patients, although 50% of these were considered grade 1 [14]. The most common late effect was a limited range of motion (moderate to severe) due to fibrosis, contracture or edema.

Studies focusing on the pediatric population (which includes the younger end of AYA patients) have noted late-term complications, including fractures, infections, bone deformities and leg length discrepancies [15]. The need for further surgeries after the resection of soft tissue sarcomas is common, and early reconstruction has been shown to mitigate some complications compared to late-stage reconstruction [16].

Surgery to remove retroperitoneal sarcomas (RPS) can be complex and require multi-visceral resection, to include concomitant nephrectomy, adrenalectomy, pancreatectomy, splenectomy, gastrectomy, enterectomy or colectomy to accomplish tumor resection. It is estimated that between 18 and 30% of retroperitoneal surgery patients experienced at least one complication requiring intervention [17,18]. Surgical risk factors for post-operative complications include the resection of more than three organs. Specifically, resection of the major abdominal veins, stomach, small bowel and pancreas carry higher morbidity compared to resections of the colon, kidney or psoas muscle [18].

With regard to the long-term effects of multivisceral resection, few studies have been performed and none specific to the AYA sarcoma population. Extrapolating from the adult data, one follow-up study showed that 49% of patients progressed to chronic stage three chronic kidney disease [19]. En bloc ipsilateral adrenalectomy is required in an estimated 50% of RPS cases. One study demonstrated that late adrenal insufficiency (AI) was found in 38.5% of patients. Using the AddiQoL questionnaire, it was noted patients with long-term AI had significantly lower scores regarding their working capacity, sweating sensations and mental concentration [20]. Pancreatic resections are notorious for early post-operative complications, but rarely complications, such as pancreatic fistula and delayed gastric emptying, can persist on the order of months [21].

### 2.2. Long-Term Morbidities from Systemic Treatment

The use of chemotherapy often plays a crucial role in controlling tumor growth and addressing microscopic disease [22]. However, as a result of this treatment, AYA cancer survivors are susceptible to a range of adverse health conditions. These health issues are often compounded by lifestyle factors and acquired health conditions, necessitating a comprehensive approach to survivorship care. Among the chemotherapeutic regimens for soft tissue sarcomas, anthracyclines, particularly doxorubicin, are frequently used either as monotherapy or in combination with alkylating agents, like ifosfamide [23,24]. While these drugs are effective in treating sarcomas, their use is associated with a broad spectrum of adverse effects that can significantly impact both short-term and long-term survivorship (Table 1).

The most severe and well-documented long term-effect of anthracycline use is cardiotoxicity, which can lead to heart failure, cardiomyopathy and inflammatory conditions, like pericarditis and myocarditis. In severe cases, death as a secondary side effect from curative intent treatment is even possible. These cardiovascular complications may not manifest until months or even years after treatment, often necessitating lifelong monitoring to manage the risk of progressive heart damage [31,32,33]. This will be discussed in detail later in this section.

In addition to cardiotoxic effects, long-term survivors of sarcoma treated with anthracyclines are at risk of renal dysfunction, peripheral neuropathy and autoimmune disorders, with complications spanning multiple organ systems [34]. One of the more pervasive complications is peripheral neuropathy, which can manifest as chronic pain, numbness, tingling and mobility issues, and it often impedes patients′ abilities to engage in everyday physical activities [33,34].

Two common classes of chronic adverse health conditions as a result of systemic treatment seen in the AYA sarcoma population are cardiovascular disease and endocrine dysfunction, which will now be discussed in detail [5,35].

#### 2.2.1. Cardiovascular Disease

Cardiovascular disease (CVD) is the leading non-cancer cause of death among cancer survivors [7]. In fact, cancer survivors are seven times more likely to die from cardiac-related events compared to their age- and sex-matched peers. This risk is especially high for females, who have a standardized mortality ratio of 8.9 compared to 6.2 for males. Cardiac radiation and anthracycline exposure are associated with an increased risk of cardiac-related mortality [36].

The precise mechanisms of the cardiotoxic effects of anthracyclines remain under investigation [37]. Studies show significant associations between the rate of doxorubicin administration and increased afterload, left ventricular dilation and impaired left ventricular function [38]. Proposed mechanisms include topoisomerase-IIβ-mediated cardiac damage and iron accumulation within mitochondria [39,40]. Chronic cardiotoxicity generally manifests within the first month, though it may arise as late as six to ten years after treatment and has an incidence rate of 1.7% [41]. Younger and older patients are particularly susceptible to developing doxorubicin-induced cardiomyopathy, with additional risk factors including a history of cardiovascular disease, female sex and higher dosing rates [37,38].

For sarcoma survivors treated with anthracyclines and/or chest radiation, appropriate surveillance is crucial. The National Comprehensive Cancer Network (NCCN) has updated their specific guidelines for the AYA population. It is recommended that survivors who are high risk (received ≥250 mg/m^2^ of anthracycline or ≥30 Gy of chest radiation) undergo echocardiographic screening every two years post-treatment and five years for those who are low risk (received ≥100 mg/m^2^ of anthracycline or ≥15 Gy of chest radiation) [5].

It is important to address long-term modifiable risk factors to manage cardiovascular disease related to sarcoma treatment. One study found that among survivors of bone and soft tissue sarcomas under the age of 40 who were treated with anthracyclines, 26% had three or more risk factors for coronary artery disease (CAD), while 49% had two or more. This prevalence is significantly higher than that seen in the general population [42]. Additionally, extremity sarcoma survivors demonstrate impaired physical fitness compared to healthy counterparts, including reduced lean mass, compromised cardiac and pulmonary function, poor balance, muscle weakness and limitations in daily activities [43].

Maintaining a healthy weight, blood pressure and heart-healthy diet are ways to protect against adverse cardiac events and should be emphasized in this population [44]. Regular exercise is encouraged due to its potential benefits; however, AYA sarcoma survivors often require structured exercise programs, as they can be tailored to accommodate each patient’s unique challenges related to the cancer location and treatment severity [7,43].

Psychological stress also contributes to cardiovascular risk [45]. Sarcoma survivors often face chronic emotional distress, which can lead to depression, anxiety and stress (discussed in detail later). This psychological burden compounds their risk of developing CVD [45,46]. A survey also found that AYA cancer survivors have a higher prevalence of current smoking compared to those who have never had cancer (26% vs. 18%) [47]. Tobacco use prevention and smoking cessation are recommended to help reduce cardiovascular risks in this population.

#### 2.2.2. Endocrine Conditions

Endocrine dysfunction is a significant concern for AYA cancer survivors. A large-scale, population-based study has found that the relative risk for endocrine disorders is 73% higher among AYA cancer survivors (including non-sarcomas) compared to their cancer-free counterparts. These survivors also face increased rates of hospitalization for endocrine disorders compared to the general population [9]. Specifically, radiotherapy for soft tissue sarcomas is associated with a 3.4 times higher risk of developing an endocrine condition after treatment [48]. Long-term endocrine side-effects span pituitary, thyroid, gonadal and metabolic dysfunction, as well as adrenal insufficiency (discussed previously).

Irradiation to the head and neck is a primary cause of central hypothyroidism and hypopituitarism. For instance, 35% of head and neck rhabdomyosarcoma survivors go on to develop a radiation-induced endocrine disorder, with 10% experiencing multiple endocrine conditions [49]. Primary acquired hypothyroidism is observed in 4% of head and neck rhabdomyosarcoma survivors within a median of 1.7 years post-diagnosis [49]. Additionally, pituitary dysfunction occurs in approximately 30% of childhood rhabdomyosarcoma survivors, with a median onset of three years after the initial diagnosis. Growth hormone (GH) deficiency is the most common of these conditions, affecting 27.5% of survivors, followed by thyroid-stimulating hormone (TSH) deficiency (9%), adrenocorticotropic hormone (ACTH) deficiency (4%) and precocious puberty (4%) [49]. Lastly, hyperprolactinemia can occur, manifesting as a decreased libido, galactorrhea and menstrual irregularities in females [44].

Gonadal functions can be vulnerable to radiation of the head and brain, as that can lead to gonadotropin deficiency, which affects puberty onset and sexual functioning in both males and females [44]. In a study of childhood rhabdomyosarcoma survivors, 2.5% developed hypergonadotropic hypogonadism within nine years post-diagnosis [49]. Radiation to the testes can result in testosterone deficiency and delayed puberty, while impaired spermatogenesis can lead to reduced fertility, oligospermia or azoospermia. Radiation directed toward the pelvic region can diminish ovarian follicular reserves, leading to delayed puberty, premature ovarian insufficiency or early menopause [44].

Chemotherapy use is also associated with endocrine-related sexual dysfunction and fertility issues, with many patients experiencing amenorrhea, oligospermia or azoospermia as a result of gonadal suppression [34]. These reproductive complications can have lasting impacts, especially for younger patients who may have concerns about their ability to have children post-treatment. This will be discussed in detail later in this manuscript.

Metabolic disorders, including diabetes and metabolic syndrome, represent significant health concerns for sarcoma survivors and compound the previously discussed cardiovascular risks. Abdominal radiation is known to affect glucose metabolism, potentially leading to dyslipidemia and diabetes [44]. A long-term study investigating mortality in sarcoma survivors revealed a significantly increased risk of diabetes-related mortality, with a standardized mortality ratio of 2.20 and the highest risk occurring 10 to 14 years after the initial diagnosis [35]. Obesity, particularly due to hypothalamic dysfunction from head irradiation, is another risk [50]. Among AYA cancer survivors, all patients have a higher prevalence of diabetes compared to those without a history of cancer (12% vs. 9%). Metabolic syndrome is also more common among AYA sarcoma survivors, with survivors aged 18 to 39 years having an odds ratio of 4.29 compared to the general U.S. population [51].

These findings highlight the critical need for treating physicians to pay close heed to reported symptoms possibly related to endocrine dysfunction, with a low threshold to initiate workup. It is essential that longitudinal care for AYA sarcoma survivors includes regular screening for endocrine dysfunction to address potential disorders that may develop following treatment, with a referral to the endocrinologist when such disorders are identified. Physicians also must consider comprehensive metabolic monitoring and intervention strategies for AYA sarcoma survivors to manage long-term health risks effectively.

## 3. Secondary Malignant Neoplasms (SMNs)

Secondary cancers are a significant long-term risk for sarcoma survivors, particularly those who have received chemotherapy and radiation, underscoring the importance of life-long surveillance [6,52]. These malignancies may present early (within the first 5 years) or take decades to appear. AYA survivors face heightened vulnerability due to the cumulative effects of their therapies over a greater duration of life-years remaining [53,54].

In a large study involving 7079 sarcoma patients, 2.2% went on to develop at least one secondary malignancy, with the highest risk observed among those treated for head and neck sarcomas. This trend likely reflects the higher rates of radiation therapy in this group, contributing to their increased vulnerability [55]. Radiation exposure increases the risk of neoplasms later in life, correlating with the regions of exposure. For example, there is a higher risk of skin carcinomas in the irradiated area. Exposure to the head and neck region increases the risk of thyroid nodules, thyroid cancer and brain tumors [35,44,52]. Chest radiation is associated with a higher risk of breast and lung cancers, while radiation to the abdomen, pelvis or spine raises the likelihood of bladder, colorectal and testicular cancers [44].

A similar study of 1499 children with primary soft tissue sarcomas found that the risk of secondary cancers spanned all histological sarcoma types, peaking within the first five years post-diagnosis. Interestingly, those survivors who developed acute myeloid leukemia did so earlier in their follow-up period, while secondary solid tumors, such as those in the bone or breast, tended to arise later [56]. Hematopoietic malignancies were the most common cause of death due to secondary malignancy in the long-term follow up [35]. Specifically, doxorubicin has been linked to the development of acute myeloid leukemia later in life [44]. The most common cause of death due to secondary malignancy in AYA sarcoma survivors is hematological malignancies (acute leukemia, chronic leukemia, Hodgkin lymphoma, non-Hodgkin lymphoma and myeloma). The majority of these deaths occur within 10 years of the sarcoma diagnosis [35].

Age at diagnosis is another factor that may assist with secondary malignancy surveillance. For example, males under 50 diagnosed with well-differentiated or dedifferentiated liposarcomas are at a particularly high risk for developing secondary malignancies. These consist of various cancer types, including myxofibrosarcoma, breast cancer prostate adenocarcinoma, clear cell renal cell carcinoma and amelanotic melanoma, to name a few [57]. Conversely for rhabdomyosarcomas, diagnosis under the age of 10 warrants more vigilant surveillance [55]. This highlights the need for age-specific monitoring and surveillance strategies for certain cancer types.

The genetic profile of a sarcoma can also influence the likelihood of secondary cancers. Fusion-positive sarcomas, which involve specific chromosomal translocations creating fusion proteins, tend to carry a lower risk of secondary malignancy compared to fusion-negative sarcomas, which lack these characteristic genetic markers and display more complex chromosomal abnormalities. Research has shown that fusion-negative sarcoma survivors face a higher risk of secondary metastasis than their fusion-positive counterparts, highlighting the role of genetic factors in long-term risk assessments [58]. Additionally, individuals with certain germline mutations—such as those with Li Fraumeni syndrome, neurofibromatosis type 1 or Rb1 gene mutation—are predisposed to elevated secondary cancer risks [56].

The germline genetics of AYA sarcoma survivors can also place patients at an increased risk for late effects [59,60]. Genetic testing is often indicated in the development of a sarcoma in AYA patients, which allows for an analysis of the germline as well. Several gene mutations have been implicated in a wide range of late effects to include cardiovascular disease, infertility, neurocognitive defects and secondary malignant neoplasms [60]. All known pathogenic variants and variants of uncertain significance (VUSs) based on germline genetic testing should prompt referral to a genetic counselor. It is important to emphasize with multi-disciplinary specialists, however, to not only monitor for secondary neoplasms based on genetic testing but to have a high index of suspicion to monitor for late effects as well.

Certain histologic subtypes are associated with distinctive secondary cancer trends. In young children, those with pleomorphic or embryonal rhabdomyosarcoma are especially vulnerable to secondary malignancies, regardless of whether they received radiation treatment, reflecting an inherent risk related to the sarcoma’s histology [53]. Dermatofibrosarcoma protuberans (DFSP) survivors, particularly females, experience a 25% greater risk of secondary malignancies, with non-epithelial skin cancers occurring at a rate over 21 times higher and soft tissue cancers five times higher than the general population. The average age at a secondary cancer diagnosis among DFSP survivors is around 41, indicating that the risk persists long after initial treatment [54]. Data on secondary cancer risk for rarer sarcomas, like undifferentiated pleomorphic sarcoma, synovial sarcoma and leiomyosarcoma, remain limited, likely due to the rarity of these cancers and the complexities associated with survival data collection in heterogeneous populations [61,62].

## 4. Psychologic Effects

While the physiologic effects of cancer and cancer treatment are important to investigate, the psychologic effects are just as important. The AYA population represents a particularly vulnerable group regarding the psychological effects of the cancer diagnosis and treatment [63]. During this crucial phase of physical, social and emotional development, the changes that accompany cancer treatment can profoundly affect one’s psyche and self-esteem. While mental health, body image and overall quality of life are inextricably intertwined, we will focus on each topic here.

### 4.1. Mental Health

Per Kosir et al., of AYA sarcoma survivors, 17 reported a change in their mental health correlated with their sarcoma diagnosis and treatment [63]. These mental health changes were often negative, brought on by concerns over mortality and feelings of guilt for not being able to perform normally. In the same group of 30 participants, 14 reported using counseling psychotherapy services. The majority reported it as a positive experience, but 4 of the 14 participants felt that the experience had inadequately addressed their psychological needs. This indicates a possible need for more targeted counseling services for AYA sarcoma patients [63].

Survivors of sarcoma have been shown to experience mood disorders at significantly higher rates than their healthy siblings [64]. Studies have shown that female sarcoma patients, in particular, report greater levels of distress compared to their male counterparts. This highlights a crucial gender difference in how these patients experience and cope with their illness [65]. For most patients, anxiety tended to peak at the time of diagnosis. As time progressed, reported anxiety gradually decreased. In contrast, depression was reported to be the most severe during the treatment phase [66]. AYA sarcoma patients report having depressive thoughts concerning their mortality and fear of cancer recurrence (FoR) throughout their survivorship journey [63].

These psychological effects of sarcoma are directly correlated with the quality of life (QoL) and symptom burden for patients. The interplay between mental and physical health demonstrates a need for comprehensive care that addresses the psychological ramifications of sarcoma alongside the physical effects [67]. Developing beneficial coping strategies is a crucial aspect for a patient’s psychological state.

### 4.2. Body Image

Patients undergoing sarcoma-directed therapy often experience side effects, like hair loss, weight fluctuations and muscle atrophy. These physical changes can severely impact one’s body image, making it a significant challenge faced by STS survivors [68,69]. Additionally, visible markers of treatment, such as scars and central venous catheters, can exacerbate adolescents’ feelings of difference and isolation from others, setting them apart from their healthy peers. STS sometimes requires limb amputation, which has further implications for survivors’ body image [70]. Patients who undergo limb amputation have been shown to experience a more negative body image [71]. Patients who have undergone limb amputation frequently cite weight gain as a negative consequence of STS treatment, largely due to reduced mobility [69].

Many young patients who suffer from low self-esteem stemming from the physical effects of sarcoma and its treatment may resort to developing negative coping mechanisms, including withdrawal from social situations and avoidance of intimate relationships [72,73]. Body image has been shown to be closely linked to sexual health and self-esteem in the general population, and this holds true for STS patients as well [74]. Those struggling with a negative body image and low self-esteem may find it particularly challenging to cultivate healthy relationships and to understand sexual health [75]. Interviews with younger STS patients reveal that concerns about body image are often intertwined with an increase in anxiety and depression [76]. As some STS survivors become more acutely aware of their bodies and the changes that accompany sarcoma treatment, this heightened awareness can also lead to increased anxiety about the FoR when noticing any difference to their body [69]. It should be noted, however, that while an STS diagnosis and treatment can negatively impact body image, in some instances, they may also inspire a more positive body image. For certain patients, the experience can motivate healthier lifestyle choices and weight loss as a result of their treatment journey [63].

### 4.3. Health-Related Quality of Life (HRQoL)

Overall quality of life following cancer treatment has been assessed in the AYA sarcoma population. The SURVSARC study was a population-based cross-sectional questionnaire study among adult (≥18 years of age) sarcoma survivors in the Netherlands [77]. In this study, AYA survivors reported significantly lower HRQoL scores across five functional areas when compared to their general population counterparts. These functional areas included physical, role, emotional, cognitive and social functioning. AYA survivors had lower scores for emotional and cognitive functioning when compared to adult sarcoma survivors. AYA sarcoma survivors reported higher rates of fatigue, pain, insomnia and financial difficulties, although these did not reach statistical significance [77].

A study examining bone and STS survivors who underwent either limb salvage surgery (LSS) or limb amputation found that those who had limb amputation reported a HRQoL score compared to their counterparts who underwent LSS [78]. The SURVSARC study demonstrated that the extremity location of a sarcoma, regardless of amputation vs LSS, resulted in low physical functioning in the AYA population compared to adults [77].

In a survey of 38 childhood, adolescent and AYA sarcoma survivors, married patients reported significantly higher HRQoL scores for vitality and mental health when compared to their non-married counterparts [79]. AYA sarcoma survivors with children also reported higher HRQoL scores with respect to physical functioning when compared to those without children [79].

## 5. Fertility and Sexual Function

Fertility and sexual function have gained significance in cancer research as studies have more thoroughly investigated the impact of chemotherapy and radiation on the human body. Both chemotherapy and radiation have been found to have detrimental effects on the fertility of male and female cancer patients, but the effects are contingent on the age of treatment, dosage of treatment and type of treatment administered [10,11]. For the AYA population, fertility and sexual dysfunction are crucial factors to consider during treatment, as they can have lasting effects on both reproductive health and the ability to maintain relationships after surviving cancer.

### 5.1. Females

The harmful effects of chemotherapy on female reproduction are primarily understood as a disruption of cell division in ovarian cells, with detrimental effects on DNA functions in these cells as well [80]. Among chemotherapeutic agents, alkylating agents have been found to be more toxic to gonadal function than other types in both men and women [81,82]. In particular, cyclophosphamide, an alkylating chemotherapy agent used in STS treatment regimens, has been found to be especially toxic to the female reproductive system. It can cause temporary or permanent amenorrhea, dose-dependent oocyte destruction, and follicular depletion [10,83]. Cumulative exposure to cyclophosphamdide resulting in ovarian failure varies by age, with older patients (>30 years) tolerating a lower dose prior to gonadal dysfunction. Recommended cumulative doses are 15–30 g total exposure for patients <20 years, <15 g exposure for patients aged 20–30 years and <10 g for patients over 30 years of age [81,84]. In a meta-analysis by Levine et al., it was found that whole abdominal or pelvic radiation ≥10 Gy in AYA postpubertal females caused greater than 80% of women to develop amenorrhea after treatment [10]. Follow-up studies of childhood cancer survivors show that approximately 8% of women develop premature ovarian failure, and this increases to 30–40% when combined with radiation [85,86].

### 5.2. Males

In males, chemotherapy and radiation can negatively impact sperm viability and production due to their gonadotoxic effects [10]. Cyclophosphamide was shown be a significant determinant in long-term sperm production in young male STS survivors, with a cumulative dose greater than 7.5 g/m^2^ showing an increased risk of permanent sterility after treatment for men as a result of damage to the spermatogenic stem cells [87]. Ifosfamide is another alkylating chemotherapeutic agent known to impact male fertility. Yonemoto et al. compared male survivors of AYA high-grade sarcoma and found that the proportion of those treated with ifosfamide who had children was significantly lower than that of their counterparts who did not receive ifosfamide, even after accounting for confounding variables. Male survivors were also significantly older than their healthy male siblings at the time of their first child’s birth, suggesting that while sperm viability can recovery after cancer treatment, there may be a refractory period before it becomes viable again [79]. Research on the effects of cancer treatment has indicated that the male sperm count is lowest during the first six months post-treatment, with temporary infertility potentially lasting up to two years [88].

The American Society of Clinical Oncology recommends health care providers discuss the possibility of infertility and the option of fertility preservation with this population of patients before treatment is initiated [89]. Interested or ambivalent patients should be referred to a reproductive specialist. Young women can consider assisted reproductive techniques, such as embryo or oocyte cryopreservation. When these are not financially feasible, there may be a benefit to ovarian suppression using GnRH analogs, although data are inconsistent regarding its benefit. Post-pubertal males may consider sperm cryopreservation when long-term infertility is a risk [89].

### 5.3. Sexual Function

Sexual function is an important consideration for AYA sarcoma survivors as they plan to build romantic relationships. Despite this, sexual dysfunction in AYA sarcoma survivors is not a widely researched topic, leaving us to apply the literature available describing the considerations of AYA patients and cancer in general. Sexual health and intimacy concerns may arise due to a complex mix of biological, psychological, interpersonal and social factors during and after treatment [12]. Direct effects from treatment, as well as indirect effects (negative body image, fatigue, depression and anxiety), may impact one’s desire for intimacy. Hormone changes as a result of gonadal toxicity may cause genitourinary syndrome of menopause or vaginal stenosis in young women resulting in dyspareunia. Neurologic toxicity may result in erectile dysfunction in young men.

The extent of surgical resection can also affect sexual function. Patients who undergo pelvic resections may experience changes in sexual function, potentially affecting both males and females [90]. Barrera et al. found that individuals who underwent amputation or Van Ness rotationplasty, rather than LSS, experienced better sexual function [91]. It is crucial to hold a comprehensive consent discussion regarding short- and long-term consequences for these patients and to mitigate such permanent side effects when possible.

The breadth, complexity and current gaps in research on this topic create challenges for AYA patients with sarcoma and their providers. Sexual dissatisfaction was experienced by nearly half of the survivors that were surveyed, around 49% in the first year after diagnosis and 43% in the second year [92]. Higher sexual dissatisfaction was also associated with the usage of chemotherapy in cancer treatment [4]. The significant prevalence of sexual dissatisfaction among AYA sarcoma survivors highlights the need for further research and support to address the physical and physiological needs of patients. Those providing oncologic care are responsible for discussing sexual health concerns at diagnosis and at regular intervals throughout survivorship [5]. If concerns are identified, referral sources may include gynecology, urology, physical therapy, psychology and sexual medicine or therapy, with interventions tailored to the unique needs of the AYA sarcoma population [5,12,93,94].

## 6. Social Issues

The AYA population is uniquely vulnerable as they navigate the challenges the accompany the cancer diagnosis and treatment, coinciding with what is already a transformative period in life. A cancer diagnosis for AYA patients can be particularly destabilizing, as it coincides with a critical period typically focused on personal and professional development. Their diagnosis and treatment impact many social areas to include marriage, career and higher education. However, there is a dearth of research regarding the AYA sarcoma patient population specifically. Here, we discuss the available data on the AYA cancer population as a whole.

### 6.1. Marriage

Among these challenges, marriage is one of the social issues that has been studied concerning AYA cancer survivors. Overall, AYA cancer survivors are less likely to be married when compared to their healthy controls, especially women [95,96]. However, when male and female AYA sarcoma survivors were compared to each other, male survivors were less likely to marry and have children than female survivors [79]. Patients who were at a younger age when diagnosed were less likely to become married [96].

This disparity in marriage rates between healthy controls and AYA cancer survivors may be explained by differences in the perceived body image and level of autonomy. Drabbe et al. found that amongst STS AYA survivors, 34% of those who experienced impaired social functioning also experienced insecurities about health-related problems that affected their romantic relationships [97].

Romantic relationships are an important source of support for AYA survivors of sarcoma. Survivors who are married score higher for their QOL compared to survivors who are not, suggesting a correlation between marital status and QOL for cancer survivors [79,98]. The disparities in marriage rates and relationship dynamics among AYA cancer survivors highlight the impact that cancer can have on well-being, emphasizing the need for targeted support to enhance their quality of life.

### 6.2. Career

The data in this area are mixed, and there are limited data specific to sarcoma patients. Some studies indicate that after treatment, AYA cancer survivors are unemployed at rates comparable to their healthy peers. One study demonstrated that AYA survivors may achieve higher education and career success but tend to begin paid employment at an older age compared to the general population [99]. Meanwhile, other studies suggest that AYA cancer survivors face higher unemployment rates at a higher rate compared to the general population [100].

The Netherland’s SURVSARC study did demonstrate that among AYA sarcoma survivors, unemployment and the need for lifestyle changes due to financial problems were associated with impaired social functioning, although this was mitigated by better social support [97]. Unemployment can mean financial hardships and little-to-no insurance coverage for any secondary or ongoing medical issues [97]. This can be a source of stress for young cancer survivors hoping to return to “normalcy” after their treatment. In a study by Bajpai et al., survivors reported anticipating stigma from co-workers or discrimination from their employer. Many jobs require chronological resumes, leaving the patients to explain a gap in employment during treatment. A fear of relapse is often listed as a reason for not finding employment [101]. The neurocognitive effects of treatment were also cited as a complication for survivors who intended to resume their previous employment but felt they could not work at the same capacity as before [102].

### 6.3. Education

Many AYA sarcoma patients receive their cancer diagnosis while pursuing secondary or higher education, leading to disruptions in their studies [103]. It can be difficult for those returning to their previous studies due to missing exams and assignments [104]. Parsons et al. found that approximately 70% of American AYA survivors returned to their previous university or workplace, but 50% reported barriers to normalcy upon their return [105]. A similar study in Denmark found that of the 50% that reported barriers upon returning to their educational institution, 60% felt that they did not receive the proper support they needed [106]. Similar to those re-entering the workforce, there may be neurocognitive effects of chemotherapy on patients that make it difficult for patients to return to the level of pre-treatment proficiency [107].

AYA sarcoma survivors may benefit from additional support services to accommodate for a gradual reintroduction into their education, including formal support services at school and support from family and guidance counselors [104,108]. Pedersen et al. describes the “drive of youth” in AYA survivors, a motivating force that powers their desire to move forward from their illness [109]. While the journey of AYA sarcoma survivors often includes a strong desire to reclaim their educational pursuits, it is crucial to recognize and address the significant barriers they face, emphasizing the need for support systems to facilitate their successful reintegration into academics.

## 7. Racial and Socioeconomic (SES) Disparities

Within the United States, race, ethnicity and insurance status have all been shown to have impacts on the treatment AYA sarcoma patients received and their subsequent survival [110,111,112,113,114,115,116,117,118,119]. Black and Hispanic patients were more likely to be in the uninsured or non-private insurance group than white patients, signaling a correlation among race, SES and prognosis [110,111]. Those with no insurance were found to be more likely to present with further advanced sarcoma or metastatic disease than those with non-private insurance or private insurance [110,111]. Tumor size at the time of diagnosis also differed between insurance groups, with 58.5% of uninsured patients having a tumor ≥ 5 cm, compared to 53.2% of patients with non-private insurance and 48.8% of patients with private insurance. In a study conducted by Penumarthy et al. at the University of San Francisco California, patients with public insurance were used as an indicator of a lower SES status. These patients were found to present with more advanced disease at diagnosis and had poorer 5-year and 10-year OS rates for AYA sarcoma patients [111].

Governmental/non-private insurance has been linked to a higher likelihood of limb amputation instead of LSS for patients with extremity sarcoma [117]. Additionally, limb salvage was more commonly associated with patients of non-Hispanic ethnicity and those from metropolitan areas [117]. For patients with late-stage, large, aggressive tumors not amenable to LSS who underwent amputation had an associated 66% increased risk of death at 10 years post-op [118]. Additionally, females were less likely to receive amputations than males to treat extremity STS [118] and were also less likely to present with advanced sarcoma compared to males [116]. Joseph et al. found that Hispanics and non-Hispanic blacks (NHBs) were less likely to undergo surgical resection when compared to non-Hispanic whites (NHWs) [116]. NHW patients were also more likely to receive radiation when compared to Hispanic and NHB patients [114].

Young NHW women are more likely to receive information regarding fertility preservation when diagnosed with cancer and follow through with the process, while non-white women are more likely to report that their provider did not mention the effects of treatment on fertility at all [120,121]. A lack of insurance was also associated with less education on fertility by patients’ providers [122]. In a study on patients who used medically indicated fertility services, Voigt et al. found that Black and Hispanic women were less likely to use fertility preservation services than White and Asian women. The average age of women accessing these services was lower for White women than every other minority group, which could possibly be explained by a delay in diagnosis for Black and Hispanic groups overall [123].

Detection of disparities outside the United States or private healthcare system do differ. In a study among universally insured sarcoma patients, racial differences in LSS and multi-modality therapy were significantly mitigated [124]. In Canada, racial disparities were mitigated for RPS treatment (not specific to AYA patients), but survival was affected by geographic location and distance to a treatment center [125]. In France, a robust reference network exists focused on sarcoma care. An analysis of patients in this network demonstrated the place of residence having no impact on sarcoma-specific survival. However, there was lower survival in “precarious population districts” in comparison to wealthy metropolitan areas, attributed to a more advanced clinical presentation at diagnosis [126].

## 8. Management of Long-Term Effects

As emphasized in this manuscript, an awareness of physiologic, psychologic and social long-term effects for AYA sarcoma survivors is essential for all providers interacting with these patients, from the primary care providers to oncologists to therapists. However, awareness without action is of limited efficacy with regards to improving the quality of survivorship. With regards to the topics covered here, we would like to summarize the recommended management and interventions for the long-term effects of sarcoma treatment.

Physiologic:

• Focused physical therapy to reduce morbidity and preserve functionality and strength for patients undergoing surgery and RT for extremity sarcoma.
• Lifelong monitoring of kidney function in patients requiring nephrectomy to monitor development of CKD in remaining kidney.
• Appreciation of possible development of late-onset adrenal insufficiency in patients who underwent adrenalectomy and appropriate screening based on reported symptoms to include fatigue and diminished mental capacity.
• Close monitoring of cardiovascular disease. Per NCCN, high-risk survivors (received ≥250 mg/m^2^ of anthracycline or ≥30 Gy of chest radiation) undergo an echocardiogram every two years. Low-risk survivors (received ≥100 mg/m^2^ of anthracycline or ≥15 Gy of chest radiation) should undergo an echocardiogram every 5 years [5].
• Deliberate prevention of cardiovascular risk factors and coaching to maintain an appropriate goal weight, blood pressure and heart-healthy diet. Smoking cessation should be strongly encouraged. Patients should partake in regular exercise, but this may need supervised modification with physical therapists depending on surgical/RT co-morbidities.
• Patients are at risk of developing a wide range of endocrinopathies. Patient-reported symptoms related to possible endocrine dysfunction should prompt an appropriate workup and referral to an endocrinologist. As some of these symptoms can be nebulous, providers treating AYA sarcoma survivors should have a high index of suspicion of endocrine disorders.
• Per NCCN, screening for secondary malignant neoplasms involves the same surveillance for the primary cancer type. Both oncologists and primary care providers should have a heightened focus on AYA sarcoma survivors due to their increased risk of secondary neoplasms depending on the treatment received.
• Patients diagnosed with genetic syndromes that predispose them to an elevated risk of sarcomas and other malignancies, such as Li-Fraumeni, neurofibromatosis type 1, Rb1 gene mutations and many others, should follow NCCN guidelines for surveillance. Often this is rigorous and includes several screening modalities, which may need to occur on an annual basis. All such patients should be referred to a genetic counselor.
• There are known genetic mutations that also predispose survivors to an increased risk of late effects. The close multi-disciplinary monitoring of these patients should occur with coordinated efforts between a clinician and genetic counselors.

Psychologic:

• AYA sarcoma survivors are at an increased risk for mental health conditions, body dysmorphia and a decreased quality of life. It is imperative for the clinician to recognize that while cancer-directed therapy may be complete, the impacts of their diagnosis are rarely “behind” them. Clinicians should have a low threshold for referral to psychologic counseling services or psychiatric specialists, even years after the diagnosis.
• Improved social support has been shown to be protective against psychologic late effects. A careful social history should be taken at each visit to assess survivor’s resources and risk.

Fertility and Sexual Function:

• Once again, the American Society of Clinical Oncology recommends that health care providers discuss the possibility of infertility and the option of fertility preservation with this population of patients before treatment is initiated [89].
• Providers should discuss the sexual function of AYA sarcoma survivors without stigma. If concerns are identified, referral sources may include gynecology, urology, physical therapy (to include pelvic floor PT), psychology and sexual medicine or therapy either for the patient individually or with their partner.

Social Issues:

• A sarcoma diagnosis in the AYA populations impacts developing romantic relationships, entering the workforce and resuming a higher education.
• Married AYA sarcoma survivors report higher HRQoL scores than their non-married counterparts. A careful social history should be taken, and if barriers to the development of stable relationships are suspected in a patient, therapy should be considered.
• There are some resources available regarding employment for survivors. In the United States, cancer survivors are protected by the Americans with Disabilities Act. Cancer and Careers and the Job Accommodation Network are two organizations in the US that specialize in reintegrating cancer survivors into the workforce. (The authors of this manuscript are not associated with either group or their sponsors.)
• Difficulties with resuming higher education are multifactorial and include issues related neurocognitive changes, physical stamina and financial implications. In most cases, the return to education should be incremental and built back to full time courses. The early involvement of academic counselors is recommended to manage academic accommodations.
• In recent years, patient advocacy groups have been formed for sarcoma survivors. In addition to being a resource for social support for survivors and their loved ones, such groups have begun to participate in clinical and survivorship research to improve short- and long-term outcomes for sarcoma patients [127].
• The role of social media with regard to social support has been studied, yielding mixed results. Based on available data, it appears to both provide a connection as well as accentuate feelings of frustration and despair. Social media should be used with caution and should not provide a primary means of support [128].

Racial and Socioeconomic Disparities

• Disparities in survival and quality of survivorship for AYA sarcoma survivors based on race, ethnicity and insurance status have been described. Providers should be aware of these disparities and work with qualified social workers to mitigate them.
• While many large cancer centers have developed AYA-specific cancer divisions, such organizations are often not available at smaller centers. Disparities may prevent some survivors from accessing resources at larger centers. Therefore, the dissemination of information, education and conversation of survivorship management and resources are paramount for all providers caring for these survivors.

## 9. Conclusions

A spectrum of survivorship considerations exist for the population of AYA soft tissue sarcoma survivors. These considerations extend beyond monitoring for cancer recurrence or known treatment co-morbidities and encompass the whole of the person, to include educational, financial, psychological and relationship factors. Optimal survivorship for this young population of sarcoma survivors will be improved by a multifactorial approach.

Sarcoma is a rare family of neoplasms, and increasing awareness within the community of those affected to participate in research will improve the quality of a field that is otherwise quite difficult to study. Strategies to improve surveillance and secondary prevention among AYA STS survivors, particularly the at-risk populations, are needed. Across all disciplines, all clinicians and therapists interacting with these patients must have a high index of suspicion for the secondary effects of the cancer diagnosis, regardless of the time since diagnosis.

## Figures and Tables

**Table 1:** Long term effects of chemotherapy used in the treatment of soft tissue sarcoma [23,24,25,26,27,28,29,30].

Chemotherapy	Known Long-Term Effects
Doxorubicin	Cardiotoxicity, myelosuppression, radiation recall reaction, erythrodysesthesia, typhlitis, peptic ulcers, and GI bleeding, renal dysfunction, peripheral neuropathy, autoimmune disorders, sexual dysfunction and infertility
Ifosfamide	Myelosuppression, GI intolerance, amenorrhea, fatigue, nephrotoxicity, secondary malignancy, neurotoxicity
Gemcitabine	Myelosuppression, peripheral edema
Docetaxel	Neuropathy, alopecia, cardiotoxicity, secondary malignancy, infertility
Pazopanib	Cardiotoxicity, depigmentation, hepatoxicity, hypothyroidism
Dacarbazine	Pulmonary toxicity, myelosuppression

## Data Availability

Not applicable.
